# Assessment of Failures of Long-acting Cabotegravir and Rilpivirine in a Real-world Treatment Setting

**DOI:** 10.1093/ofid/ofaf349

**Published:** 2025-06-17

**Authors:** Nicholas Yared, Smitha Gudipati, Shannon Payne, Indira Brar

**Affiliations:** Henry Ford Health, Department of Medicine, Division of Infectious Diseases, Detroit, Michigan, USA; Henry Ford Health, Department of Medicine, Division of Infectious Diseases, Detroit, Michigan, USA; Henry Ford Health, Department of Medicine, Division of Infectious Diseases, Detroit, Michigan, USA; Henry Ford Health, Department of Medicine, Division of Infectious Diseases, Detroit, Michigan, USA

**Keywords:** cabotegravir, HIV, rilpivirine, virologic failure

## Abstract

Fifty-eight people with HIV switched to long-acting cabotegravir and rilpivirine in a real-world clinic setting had higher discontinuation rates because of virologic failure, side effects, or nonadherence compared to clinical trials. Archived proviral genotype testing before long-active cabotegravir and rilpivirine switch should be considered to reduce virologic failure risk.

The first complete long-acting (LA) injectable antiretroviral therapy (ART) consisting of the second-generation integrase strand transfer inhibitor (INSTI) cabotegravir (CAB) and the nonnucleoside reverse transcriptase inhibitor (NNRTI) rilpivirine (RPV) was approved by the US Food and Drug Administration in January 2021 for maintenance treatment in people with HIV (PWH) [1]. The virologic efficacy of LA CAB/RPV has been assessed in multiple randomized clinical trials, showing noninferiority compared to daily oral INSTI-containing ART [2–8]. Although its occurrence is rare, the etiology of virologic failure (VF) during LA CAB/RPV merits further evaluation. VF can have significant impacts on future treatment options due to the selection of drug resistance mutations (DRMs) as managing PWH who experience ART failure can be complex. Evaluation of failure of a drug or drug combination to achieve undetectable HIV viral load (VL) should assess both virologic efficacy and drug tolerability. Drugs that are not well tolerated lead to nonadherence and pose a challenge to medical management of HIV infection. In this study, we describe the inability to achieve an undetectable HIV-1 VL with LA CAB/RPV in a diverse group of patients in a real-world setting.

## METHODS

This was a retrospective cohort study conducted from August 2021 to March 2023 at Henry Ford Health in Detroit, Michigan. Inclusion criteria included all PWH suppressed on ART who had initiated LA CAB/RPV with 12-month viral loads available after starting therapy. LA CAB/RPV failure was defined as the regimen being stopped after greater than 1 dose because of VF or side effects. VF was defined as a VL ≥ 30 copies/mL on 2 occasions greater than 4 weeks apart. Demographics, clinical characteristics associated with VF, and genotype-related DRMs were collected from the electronic medical record.

Demographics and clinical characteristics were analyzed using frequencies for categorical variables and means with standard deviation (SD) for continuous variables. Genotype-related DRMs performed through next-generation sequencing included mutations with greater than 1% of the population frequency. Minority DRMs were defined as DRMs detected at < 10% frequency.

## RESULTS

A total of 58 individuals initiated LA CAB/RPV between August 2021 and March 2023. Patient demographics are shown in Table 1. The mean age of the cohort was 47 years (SD: 14 years). A majority of patients were male (n = 48, 82.8%) and identified as Black (n = 43, 74.1%). Patients who were obese with a body mass index (BMI) ≥ 30 made up more than half of the cohort at 51.7% (n = 30). The cohort was significantly treatment-experienced with patients with HIV an average of 17 years (SD: 9.2 years), 31 (53.4%) having NNRTI exposure, and 44 (75.9%) having INSTI exposure before the switch. The mean CD4+ T-cell count at the time of switch from oral ART to LA CAB/RPV was high at 845 cells/µL (SD: 306 cells/µL). At 12 months following initiation of LA CAB/RPV, 50 patients had VL data available, with 49/50 (98%) having HIV-1 VL < 30 copies/mL. The patient with a detectable HIV-1 VL at 12 months had an increase in HIV-1 VL to 530 copies/mL following prior undetectable values at baseline, 3 months, 6 months, and 9 months.

**Table 1.** Demographics and Clinical Characteristics

Variable	Frequency (n = 58)
Age (y)^a^	47 (14)
Gender, n (%)	
Male	48 (82.8%)
Female	10 (17.2%)
Race/ethnicity, n (%)	
Black	43 (74.1%)
White	14 (24.1%)
Other	1 (1.7%)
Body mass index (kg/m^2^)^a^, n (%)	
< 25	9 (15.5%)
25–29.9	19 (32.8%)
≥ 30	30 (51.7%)
Years with HIV^a^	17 (9.2)
NNRTI exposure before switch^b^, n (%)	31 (53.4%)
INSTI exposure before switch^c^, n (%)	44 (75.9%)
CD4+ T-cell count at switch (cells/µL)^a^	845 (306)
HIV-1 viral loads (copies/mL), n (%)	
At time of switch	
Not detected	53 (91.4%)
Detected (≥ 30 copies/mL)	5 (8.6%)
At 12 mo^d^	
Not detected	50 (98%)
Detected (≥ 30 copies/mL)	1 (2%)

Abbreviations: INSTI, integrase strand transfer inhibitor; NNRTI, nonnucleoside reverse transcriptase inhibitor.

^a^Age, years with HIV, and CD4+ T-cell count at switch are given as means with standard deviation in parentheses.

^b^NNRTI exposure for 1 patient was unknown.

^c^INSTI exposure for 1 patient was unknown.

^d^Eight patients had not had a 12-month HIV-1 viral load checked.

Among the 49 patients retained on LA CAB/RPV therapy at 12 months, 47 had further VL data available at 24 months with 45 patients continuing to receive LA CAB/RPV. Of the 2 patients who had discontinued LA CAB/RPV between 12 and 24 months after initiation, 1 patient was lost to follow-up because of moving to another state and the other became pregnant with the decision made to discontinue LA CAB/RPV during her pregnancy. No additional patients who received LA CAB/RPV for greater than 12 months were found to develop VF requiring while on LA CAB/RPV.

Of our patient sample, 9/58 (15.5%) discontinued LA CAB/RPV before the 12-month mark. Among patients discontinuing LA CAB/RPV, 3/9 (33.3%) met VF criteria and 2/9 (22.2%) developed severe injection site reactions. Additionally, 4/9 (44.4%) patients discontinued LA CAB/RPV for reasons other than VF or side effects. These reasons included nonadherence, with LA CAB/RPV leading to a switch back to oral ART (n = 2), missing an LA CAB/RPV dose because of hospitalization with subsequent change to oral ART (n = 1), and administrative discharge from the healthcare system (n = 1).

Genotype data for the 3 patients with VF is shown in Table 2. The mean time of having HIV among patients with VF was 19 years (SD: 9 years), and all had a BMI ≥ 30 kg/m^2^. All 3 patients experiencing VF on LA CAB/RPV had maintained on-time injections with 1½ inch needles. All patients had HIV subtype B.

**Table 2.** NNRTI and INSTI Resistance Mutations in Patients Experiencing Virologic Failure on Injectable Cabotegravir and Rilpivirine^a^

Patient	Pretreatment Genotypes (GTs)^b^		Posttreatment Genotypes (GTs)^a^	
	NNRTI Mutations	INSTI Mutations	NNRTI Mutations	INSTI Mutations
1	Not performed	Not performed	8/28/23 GT	8/28/23 GT
			E138 K (8.5%)	E92K
			M230I	Q146R
			12/22/23 GT	12/22/23 GT
			E138E/K	None
			M230L	
2	Not performed	Not performed	1/26/23 GT	1/26/23 GT
			Y188L	E138K
			V106I	Q148K
3	1/13/22 GT	1/13/22 GT	6/13/23 GT	6/13/23 GT
	None	G140R (1.2%)	E138K	Q148R

Abbreviations: INSTI, integrase strand transfer inhibitor; NNRTI, nonnucleoside reverse transcriptase inhibitor.

^a^All patients experiencing virologic failure had HIV-1 subtype B.

^b^Mutations found as minority drug resistance mutations (detected at frequency < 10%) have their frequency shown in parentheses.

One patient had a pretreatment genotype that showed a minority G140R INSTI DRM at a prevalence of 1.2%. This patient was found to have an increase in HIV-1 VL to 2444 copies/mL at 3 months after initiation of treatment with development of the E138 K NNRTI DRM and Q148R INSTI DRM. Baseline genotypes before initiation of LA CAB/RPV were not available for the 2 other patients experiencing virologic failure. One patient was found to have a 12-month HIV-1 VL of 530 copies/mL, and a standard genotype showed the M230I NNRTI DRM, E138 K minority DRM at a prevalence of 8.5%, and E92 K and Q146R INSTI DRMs. The other patient was noted at 3 months to have an increase in their HIV-1 VL to 45 011 copies/mL. Genotypic testing showed the development of the V106I, I135I/T, and Y188L NNRTI DRMs and the E138 K and Q148 K INSTI DRMs. All 3 patients had their regimen changed to the coformulated single-tablet oral regimen tenofovir alafenamide/emtricitabine/darunavir/cobicistat based on genotype results.

## DISCUSSION

Among 58 patients undergoing switch from oral ART to LA CAB/RPV, 9 (15.5%) discontinued LA CAB/RPV by 12 months because of either VF, side effects, or nonadherence. This is in contrast to the 12-month data from the ATLAS trial that showed only 2% of per-protocol patients experienced LA CAB/RPV failure by 12 months. Moreover, patients discontinuing CAR because of site injection reactions comprised 3.4% of our population, which was higher than that seen in a pooled analysis of LA CAB/RPV from the phase 3 ATLAS and FLAIR trials (1% discontinuation) [1]. In our study, 4 patients discontinued LA CAB/RPV because of regimen nonadherence and missing on-time injections because of hospitalization. These issues prompted a switch back to oral ART, which none of the clinical trials addressed. Additionally, no further development of virologic failure was seen in patients continuing LA CAB/RPV after remaining on LA CAB/RPV for greater than 12 months with discontinuation related to reasons other than VF.

Among the 3 patients who met the criteria for VF, genotypes revealed the development of 2-class resistance (NNRTI and INSTI DRMs). Of note, the E138 K mutation was seen in all posttreatment genotypes with 1 patient having both the E138 K in combination with the Q148 K mutation for the INSTI ART class. Among the 3 patients with VF, they had had HIV for a longer period compared to the overall group. While we were diligent in extracting all genotype data before initiation of LA CAB/RPV, not all treatment-experienced patients who were diagnosed decades ago had had baseline genotypes performed or available. Our findings of the development of INSTI DRMs are similar to a recent paper by Navarro et al., who reported INSTI resistance in 40%–70% of individuals experiencing VF (1%) with LA CAB/PRV through a systematic review of all studies reporting CAB/PRV VF [9]. Given the extensive use of NNRTIs as part of ART in the early twenty-first century before the implementation of INSTIs as preferred regimens, we hypothesize that patients experiencing VF may have had archived NNRTI resistance that was unknown or not seen on prior standard genotypes. Another factor possibly contributing to the development of VF was that all 3 patients were obese with a BMI ≥ 30 kg/m^2^, and our clinic was initially using 1½-inch needles for administration of LA CAB/RPV in all patients irrespective of their BMI. Orkin et al. also described the presence of VF with BMI ≥ 30 kg/m^2^; however, this was in combination with either RPV DRMs or A6/A1 subtype [10]. Our patients had HIV subtype B, and not all of the individuals had predetermined RPV DRMs, but subsequently developed them.

Of note, in a previous study the presence of at least 2 rilpivirine DRMs picked up by proviral DNA genotypes has been shown to have a statistically significant association with development of VF for patients receiving LA CAB/RPV [4]. Given the difficulty of obtaining historic genotype data for PWH who may have been diagnosed many years ago and had been on multiple prior regimens containing NNRTIs, proviral DNA genotypes may be considered to look for archived DRMs in heavily treatment-experienced PWH before switch to LA CAB/RPV. However, because of issues with the sensitivity of proviral DNA genotypes, results should always be evaluated based on knowledge of a patient's treatment history to provide clinicians with the best information to determine the risk of VF with a switch to LA CAB/RPV. Genotypic information is not always available in resource limited settings and can be challenging to obtain. The CARES trial enrolled virally suppressed individuals with switch to LA CAB/RPV with no prior resistance testing and found LA CAB/RPV to be noninferior to oral therapy at 48 weeks [11].

Future LA agents currently under study also offer the possibility of use in combination with LA CAB/RPV for patients wanting to switch to an all-injectable regimen but who may have DRMs that prohibit use of LA CAB/RPV by itself. However, more studies looking at the combination of LA CAB/RPV with other LA agents are needed.

## CONCLUSION

Among the first 58 PWH switching from oral ART to LA CAB/RPV in our clinic, discontinuation rates were higher compared to clinical trials and occurred secondary to virologic failure, injection site reactions, nonadherence, and inability to receive the drug while hospitalized. Performance of archived genotype testing in heavily treatment experienced PWH, especially those that have no prior genotypes available, should be considered before switch to LA CAB/RPV to reduce risk of VF.

## Notes

***Acknowledgments.*** The authors thank our patients with HIV who we take care of in our clinic as well as the support staff at Henry Ford Health.

***Author Contributions.*** N.Y., S.G., and I.B. contributed to the study design, data collection, data analysis, writing the manuscript, and editing the manuscript. S.P. contributed to data collection and editing of the manuscript. All authors approved the final version of the manuscript.

***Patient consent statement.*** The study protocol was approved by the Henry Ford Health institutional review board.
